# The COVID-19 Army: Experiences From the Deployment of Non-Hospitalist Physician Volunteers During the COVID-19 Pandemic

**DOI:** 10.1017/dmp.2021.109

**Published:** 2021-04-06

**Authors:** Kevin D. Hauck, Katherine A. Hochman, Mark B. Pochapin, Sondra R. Zabar, Jeffrey A. Wilhite, Gretchen Glynn, Brian P. Bosworth

**Affiliations:** 1 Department of Medicine, New York University Grossman School of Medicine, New York, NY, USA; 2 New York City Health and Hospitals, New York, NY, USA

**Keywords:** delivery of health care, emergency medicine, emergency preparedness, health care facilities, safety management, services, workforce

## Abstract

**Objective:**

New York City was the epicenter of the outbreak of the 2019 coronavirus disease (COVID-19) pandemic in the United States. As a large, quaternary care medical center, NYU Langone Medical Center was one of many New York medical centers that experienced an unprecedented influx of patients during this time. Clinical leadership effectively identified, oriented, and rapidly deployed a “COVID Army,” consisting of non-hospitalist physicians, to meet the needs of the patient influx. We share feedback from our providers on our processes and offer specific recommendations for systems experiencing a similar influx in the current and future pandemics.

**Methods:**

To assess the experiences and perceived readiness of these physicians (n = 183), we distributed a 32-item survey between March and June of 2020. Thematic analyses and response rates were examined to develop results.

**Results:**

Responses highlighted varying experiences and attitudes of our frontline physicians during an emerging pandemic. Thematic analyses revealed a series of lessons learned, including the need to (1) provide orientations, (2) clarify roles/workflow, (3) balance team workload, (4) keep teams updated on evolving policies, (5) make team members feel valued, and (6) ensure they have necessary tools available.

**Conclusions:**

Lessons from our deployment and assessment are scalable at other institutions.

## Introduction

New York City was the epicenter of the 2019 coronavirus disease (COVID-19) pandemic in the United States, with over 92 000 hospitalizations and 29 000 confirmed deaths to date.^1^ As a large, quaternary care medical center in New York, NYU Langone Medical Center was one of many New York medical centers that experienced an unprecedented influx of patients during the COVID-19 pandemic. The medicine census (intensive care unit [ICU] and acute) increased from a maximum of 200 patients to a peak of 550. The number of acute medicine teams increased from 12 during normal operations to a maximum of 29 over 3 weeks. To care for these patients, we formed a “COVID Army” – providers from medicine, surgery, and other specialties who volunteered to work on the inpatient wards during the surge. To strategize and operationalize change, we created the nimble COVID Army working group consisting of operational and clinical leadership to effect the following fivefold strategy:

1. **Identify faculty:** Using REDCap, we surveyed the entirety of the NYU Langone clinical faculty for willingness to volunteer, ability to lead a ward team and/or an ICU team as well as the presence of an authorized medical exemption (including age). Faculty who participated opted to do this instead of outpatient telemedicine or other assignments, and were not forced to participate if they felt unable.
2. **Categorize responses:** Using hospital operations administration support, we created a master database that collated these responses and allowed for quick reference and schedule mapping.
3. **Assess developing needs**: By constantly staying abreast of the volume and severity of patients during the surge, often on a thrice daily basis, the COVID Army working group undertaking was facile in opening and closing medical units and deploying/re-deploying to meet the clinical needs.
4. **Orient new teams:** Managing the onboarding of new teams was accomplished via a 1-hour Webex session focusing on infection control, inpatient use of the electronic health record (EHR), and a re-introduction to hospital medicine. Additional explanation of use of personal protective equipment (PPE) was provided through pre-recorded videos and in-the-moment teaching led by hospitalists and advanced practice providers. Additional supervision was provided by hospitalist team leads and hospitalist unit directors.
5. **Deploy faculty:** By partnering with departmental and divisional leadership, the COVID Army working group was able to recruit faculty volunteers from all over the health system. These faculty were deployed as needed and functioned either as primary ward attendings or as supplemental hospitalists operating under a lead hospitalist. One deployment consisted of 7 days of work over a 2-week period.

Although prior literature has described general principles of hospitalist staffing in the time of COVID-19,^2-4^ how to theoretically leverage specialists,^5^ and adaptations to hospital wards,^6^ little has been written on the experiences of the COVID Army, those physicians from multiple specialties who were mobilized to care for this first surge of patients.

Outcomes for patients with COVID-19 at our institution have also been previously described.^7,8^ The goal of this study is to share feedback from our frontline providers on our capacity building process and to use information gathered to offer specific lessons learned in planning for future outbreaks.

## Methods

To assess physicians’ attitudes and feedback while working in the COVID Army, a 32-question survey on 3- and 5-point Likert scales was designed by clinical leadership and administered via Qualtrics from March–June 2020. Faculty were surveyed immediately upon completion of their rotation. Questions included provider demographics and an assessment of attitudes toward their experience on their COVID team. Opened-ended questions further elicited provider experience during the outbreak. Data were collected without identifiers to protect anonymity. Individual-level consent was not required as distribution and analysis of the survey qualified as a quality improvement project through NYU’s institutional review board.

## Results

All 272 faculty physicians who volunteered to work in the COVID Army received a survey. Of these, 67% (n = 183) responded. The demographics of these respondents are captured in Table 1. Of the respondents, 84 (46%) were from the Department of Medicine and the remainder were primarily from surgical, pediatrics, or obstetrics/gynecology specialties. Respondents worked in combination ambulatory/inpatient practices (n = 94; 52%) or outpatient only (n = 85; 47%). The mean number of years in practice was 7.18.

**Table 1.** Demographics

Item	Response Options	n
Primary Department (n = 183)	Medicine	84
	Orthopedic Surgery	35
	Other	13
	Surgery	9
	Urology	8
	Ophthalmology	7
	Plastic Surgery (Hansjörg Wyss)	6
	Obstetrics and Gynecology	5
	Population Health	4
	Dermatology (Ronald O. Perelman)	2
	Neurosurgery	2
	Transplant Surgery	2
	Radiology	2
	Pediatrics	1
	Emergency Medicine	1
	Rehabilitation Medicine (Rusk Rehabilitation)	1
	Health Sciences Library	1
Division within Department of Medicine (of those with primary department as Medicine) (n = 62/183)	Gastroenterology	16
	General Internal Medicine and Clinical Innovation	16
	Endocrinology, Diabetes, and Metabolism	8
	Cardiology (Leon H. Charney Division of Cardiology)	8
	Geriatric Medicine and Palliative Care	5
	Hematology and Medical Oncology	4
	Rheumatology	4
	Infectious Diseases and Immunology	1
Pre-COVID-19 role (n = 183)	Faculty	168
	Fellow	10
	Other	5
Mix of practice pre-COVID-19 outbreak (n = 181/183)	Both inpatient and outpatient	94
	Amb/outpatient only	85
	Inpatient only	2
FGP or voluntary (n = 163/183)	FGP	147
	Voluntary	13
	Other	3
Unit acuity (assignment) (n = 165/183)	Acute	93
	ICU drop down	38
	Other	34
Role on COVID Team (n = 170/183)	Supplemental attending	111
	Primary hospitalist	42
	Other	12
	Fellow (as primary hospitalist)	5

*Notes:*

FGP = faculty group practice; ICU = intensive care unit.

A total of 111 respondents worked as supplemental hospitalists, whom we defined as physicians serving the role as interns or advanced-practice providers supporting the attending hospitalist; 45 worked as primary hospitalists. On a 3-part Likert scale (*Too few, just right, too many*), 130 respondents (76%) felt that the number of patients they were in charge of felt “just right.” On a 5-part scale (*Not at all, a little bit, somewhat, very, completely challenging*), 10% rated the experience as very challenging or challenging (n = 17). On their perception of support and training, 94% and 63% rated the support they received and training they received as “somewhat” or “very effective,” respectively (scale: *Not at all, only a little, somewhat, very effective*); 89% (n = 99) and 96% (n = 107) of supplemental attendings felt valued and valuable to their team, respectively; 87% of respondents identified as being willing to volunteer for the next week as needed.

Thematic analysis of qualitative feedback reinforced quantitative experiences (Table 2). Themes identified included a sense of comradery and community, as well as the need for investment in detailed orientations, enhanced training on EHR use, provision of proper tools (ie, protective equipment or locker space), and the need for clarifying roles, balancing workloads, and keeping teams abreast to evolving policies. Almost all qualitative comments mentioned the strong connections that the clinicians felt to one another, their team, patients, and the hospital system. A strong sense of team comradery and individual value were mentioned specifically in most comments.

**Table 2.** Recommendations and lessons learned with qualitative data

Themes	Recommendations/Lessons Learned	Provider Feedback on Areas for Improvement*
**Training and Orientation**	Invest time into orientations, include more training on EHR use with at-the-elbow assistance.	*Practice modules for EPIC – orders, admissio ns, discharges, etcetera, as this took a lot of time.* *I think a video introduction to the system would have been helpful, especially for those of us not in the Department of Medicine, and not familiar with in-patient care.* *Would also be helpful if their orientation included written material that they can refer to regarding how to discharge, how to discharge to LOH, etcetera. It may already include that, but they vocalized not having it in orientation.* *I was able to better understand how to use aspects of the Epic that we do not usually use in the ambulatory care center. I learned about the Epic chat features. Became more in touch with my internal medicine which came back from medical school.* *I’m hands-on, so it’s difficult for me to learn by just following a video. Perhaps an exercise where you have to do a progress note as well as daily orders, new admission orders, and discharge orders.* *Would suggest a video that would go through the day, what your responsibilities are, and how to handle them. Would outline the Epic tools you need, especially the admission order sets, and charting, as this is what most time was spent on.* *…more granular[ity] regarding how to transfer a patient and clinical criteria for each of the units, etcetera. Also, clear ways of clinical elevation (how to reach alert team and then ICU), a lot of people coming on are not used to calling consults.* *Better training – my co-volunteer did not have a good sense of the role of PPE or infection control, and made me very apprehensive about possible spread as that individual was not following the guidelines.*
**Roles and Teams**	Clarify roles and workflow within each team upfront, and clearly identify lines of escalation.	*A description of each assignment might be useful. If you were able to group floors and explain what they were, that would help those at home and those not clear what they might be asked to do.* *Provide more information on roles and responsibilities. We were given minimal information on workflow and expectations.* *Identify a hospitalist to consult for clinical issues each week.* *Worked with an outpatient provider who hasn’t been in the [inpatient] environment for years and so it was difficult for her/him/them to do these intern tasks. So, ultimately, I ended up taking more patients, which gave me a 7-patient load. And of course, they all started decompensating around the same time, which was difficult to manage. I think it would be good to emphasize to those who are volunteering that they’ll be functioning as an intern again or at least delineate the responsibilities prior to day 1 of work, because the title of “supplemental attending” can be misleading.* *I think the coordination of teams would be most important before starting, and I do recognize how challenging that is in the midst of a pandemic! However, I was a primary hospitalist transitioning mostly to non-COVID patients (after reading up on everything for COVID for several days!) and haven’t done inpatient medicine in 10 years. I thought I would have a resident, or at least an NP or PA that knew Epic so I could focus on the medicine….That being said, the support was great to figure out problems in real time and I relayed that in the end.* *Pair hospitalists with those of us who were hospital naïve. Perhaps circulate some guidelines to care – how often to get inflammatory markers? What to do with anti-coagulation?*
**Clinical Load**	Balance team workload if possible.	*Balance acuity between teams, or make the high-acuity services smaller (less patients). My unit was essentially a 17-bed step down unit with every patient on continuous monitoring and many on high flow nasal cannula.* *One thing to consider is staggering supplemental staff, so everyone is not new at the same time. The person that has been around for a day or two could teach the new team members.* *Triage volunteered skills/experiences better to allow for redeployment that optimizes volunteers’ abilities, which would make for a more enjoyable experience for the volunteer as well.* *Patient care was smooth and streamlined – everyone understood their role and there were no issues getting the patients the care they needed, support for discharge when indicated, and keeping communication open with their families.*
**Guidance**	Keep teams updated on evolving policies and recommendations, with focused and clear guidelines.	*More support/explanation regarding investigational treatment options for patients.* *A little more guidance in terms of newest information, but was able to figure it out quickly.* *Would be very helpful to have a one-page handout or website to refer to regarding the current trials, criteria for using various medications, consideration of anticoagulation. For example, I thought the one-page flowchart of the anticoagulation was supremely helpful… would have been nice to have that readily available on a central site along with other useful information. If such a site exists, would be helpful to include that on the orientation e-mail.* *The algorithms for COVID care that we were given in our initial e-mails were very helpful, although seemed slightly outdated to what was being practiced on the floors.*
**Support**	Make team members feel valued. Provide clear lines of proactive support from hospitalists and intensivists.	*There was variety in amount of oversight/support by hospitalists. I appreciated the hospitalist checking in often.* *The emotional support and gratitude from all were heartfelt and so gratifying. The patients were also so appreciative for what was being done in these most trying times. The hospital support services are awesome. I’m sure this is something in place at all times but to have the support in this environment was much appreciated.* *I liked having a team and people above me to turn to. It was nice to feel a part of the overall care group and feel involved with what’s going on.* *The oneness I felt with all the health care team made the experience quite a remarkable one. I feel the support everyone gave each really did help save the lives of so many people.* *The tone in the hospital was overall more collaborative and willing to help, especially consulting services which was really nice to see.* *I felt very valued at every step of the way. They could not have been more welcoming or thankful for my help.* *It was really great to get to know people from other departments. We all felt like fish out of water a little, and it was really great to support each other and learn together. Also, I felt rewarded to do my part in taking care of patients and fighting this terrible virus.* *Being there for patients. It was good to have a team focused on communicating with the families – they were wonderful!* *The tone in the hospital was overall more collaborative and willing to help, especially consulting services which was really nice to see. The actual patient interactions were the most rewarding – I would have loved to spend more time bedside (and less doing the admin charting, etcetera!).*
**Resources**	Ensure they have the right tools available, with an emphasis on must-know information.	*We need a handbook with phone numbers and quick how-to’s.* *A printable “cheat sheet” could have been useful for sign in, med rec, discharge med rec, finding MAR, and a brief dashboard setup to make finding things like labs, etcetera, easier.* *…Lockers, more scrubs, a cleaner and less crowded physician workspace would have been useful.* *Wish we did not have to reuse N95 masks, regular surgical masks and head coverings.*

*Note:*

* All pronouns in the written comments changed to include both masculine and feminine pronouns to ensure confidentiality (respondents used perceived gender in their comments).

## Discussion

In this paper, we summarize the experience of the COVID Army, those physicians from non-hospitalist specialties who volunteered to work as hospitalists and supplemental hospitalists. These data demonstrate that, with nimble leadership, it is feasible to rapidly identify, assess capabilities, orient, and deploy physicians from very diverse departments and practice settings. The COVID Army effectively cared for 1689 COVID-19 patients during the initial surge from March 30 to May 30, with a majority identifying as willing to join the cause again in the future.

As the country prepares for the risk of prolonged community transmission, it is imperative that we harness lessons learned. Most importantly, we demonstrate that the majority of COVID Army volunteers felt valued and valuable, and would be willing to volunteer for the next wave. Many wrote in the survey that the experience was meaningful and that they would want to join another round of assignments as part of the COVID Army. Others are still talking about the community experience. We accomplished this with transparent leadership, frequent communication, and a spirit of comradery.

It was critical for leadership to develop a strategy to be both transparent and connected, despite not being physically present. To this end, we created a daily newsletter, *The COVID Daily*, which was sent via e-mail every night to the entire COVID army, hospital leadership, and all the inpatient disciplines (social work, care management). This daily communication included the latest metrics, updates in the staffing and patient composition by unit, the numerous clinical trials, as well as COVID-19-related best practices and resources. In addition, we created a virtual twice weekly COVID continuing medical education (CME) lecture series in which primary investigators presented key clinical trials, thereby ensuring all providers understood what drugs were available to patients. This allowed those caring for COVID-19 patients the ability to enroll patients in clinical trials and thus contribute to the understanding of the disease. Further, hospitalists led a bi-weekly, rapid-fire journal club in which key studies were reviewed. Other lectures included an autopsy review, a neuroradiology update, COVID-19 illnesses by system, a history of plagues through the ages, and a leadership series. The audience size for these lectures was around 75 people, on average. From March to August, over 1500 CME hours were issued. Last, in order to promote wellness, we offered a monthly hospital-wide book club open to all. For our first selection, over 75 people joined 1 of 5 sessions, which suggested an overarching need for participants to connect with peers.

During this period, we regularly conversed with our frontline staff who were struggling with updating families of COVID-19 patients, especially since visitors were no longer allowed to visit due to the NY State imposed stay-at-home order.^9^ In response to this hardship, we created an innovative program called *NYU Family Connect* in which the families of every COVID-19 patient received a call from a medical professional.^10^

While there are no prior data on appropriate number of patients in pandemics, our data suggest an average of 7 patients per hospitalist extender to be an appropriate target. Future iterations could allow the number of patients to vary, depending on the specialty of the participant. Recent graduates from medical subspecialties or surgical specialties may be better positioned to care for more patients.

Most participants felt that training was sufficient, but comments revealed frustration with the electronic medical record (EMR). This is not surprising as many physicians struggled with simple tasks like setting up their inpatient lists and accessing the COVID-19 treatment screen. While the most important EMR areas were covered in orientation and provided in a handbook, this virtual training was not adequate. Open-ended responses from a small portion of respondents also reflected mixed attitudes toward the amount of PPE, with most noting that this issue was beyond hospital control. Other concerns over resources included concerns of personal storage space and occasional uneasiness over workstation cleanliness.

There was confusion around how exactly a hospitalist extender would interface with the hospitalist and the rest of the interdisciplinary team. This was exacerbated when the hospitalist extenders were chairs of departments.

## Conclusion

Given what we have learned from our survey and the unknown length of the pandemic and potential for future waves, we continue to provide transparent leadership and frequent communication with a nightly informative and inspirational e-mail and an educational series to ensure that everyone is learning together. This also provides a sense of community. We also ensure that families unable to visit will be informed of regular updates. We believe that 7 COVID-19 inpatients is the appropriate number of patients to be assigned to each physician extender, although we recognize that this number may increase since we have more comfort in caring for COVID-19 patients now compared to previously.

Upon reflection, there are several components we would change, including how we train physicians to use the EMR. Rather than doing so during a virtual orientation, we would provide more help “at the elbow.” In addition, we would be more specific in defining exactly the role of the hospitalist extender and clarifying lines of escalation. In the interim, we have created a comprehensive orientation guide with hyperlinks to various protocols and studies to ensure that physicians have up-to-date information. We hope our system will never again see the numbers we did and believe that with the evolving knowledge of the clinical course of coronavirus, increased testing and vaccination in NYC, and lessons learned, we are now prepared to increase our capacity and efficacy, and to effectively use all we have learned.
